# Advanced Dye Sorbents from Combined Stereolithography 3D Printing and Alkali Activation of Pharmaceutical Glass Waste

**DOI:** 10.3390/ma15196823

**Published:** 2022-10-01

**Authors:** Mokhtar Mahmoud, Jozef Kraxner, Hamada Elsayed, Dušan Galusek, Enrico Bernardo

**Affiliations:** 1 FunGlass, Alexander Dubček University of Trenčín, 911 50 Trenčín, Slovakia; 2 Department of Industrial Engineering, University of Padova, 35131 Padova, Italy; 3 Department of Glass Research, National Research Centre, Cairo 12622, Egypt; 4 Joint Glass Centre of the IIC SAS, TnUAD and FChFT STU, 911 50 Trenčín, Slovakia

**Keywords:** dye sorbents, alkali activation, glass waste, 3D printing, SLA-stereolithography

## Abstract

Additive manufacturing (AM) technologies enable the fabrication of objects with complex geometries in much simpler ways than conventional shaping methods. With the fabrication of recyclable filters for contaminated waters, the present work aims at exploiting such features as an opportunity to reuse glass from discarded pharmaceutical containers. Masked stereolithography-printed scaffolds were first heat-treated at relatively low temperatures (680 and 730 °C for 1 h) and then functionalized by alkali activation, with the formation of zeolite and sodium carbonate phases, which worked as additional adsorbing centers. As-sintered and activated scaffolds were characterized in terms of the efficiency of filtration and removal of methylene blue, used as a reference dye. The adsorption efficiency of activated printed glass was 81%. The 3D-printed adsorbent can be easily separated from the solution for reuse.

## 1. Introduction

Glass recycling is far less straightforward than it appears [1,2]. The use of a cullet as a feedstock for the fabrication of original articles by remelting cannot always be applied [3,4]. Some glasses, in fact, are ‘unrecyclable’ for several reasons, including the risks of degradation of their properties or the loss of chemical purity [5]. The main obstacle to reusing glass waste is that it should pass through various expensive and time-consuming steps [6,7]. Therefore, the obstacles involved in the upcycling of glass waste for glass manufacturing, coupled with its growing quantities and non-biodegradable nature, bring the need for the development of new applications [5]. The latter aspect is particularly important in glasses for pharmaceutical containers, normally fabricated from high-purity minerals and shaped into preforms (e.g., tubes and rods, to be later transformed into vials, syringes, etc.) in highly specialized plants [8,9,10]. The situation is complicated by the current COVID-19 emergency, greatly enhancing the production of packaging (e.g., destined for vaccines) and, obviously, of related waste [11].

The abovementioned ‘unrecyclability’ actually refers to a closed-loop model. However, discarded glasses may also be reused in the manufacturing of new products, in different application domains, according to an open-loop model. These new products are sustainable if the commercial value, in structural and functional applications, compensates for the costs of the transformation operations [12,13]. Viscous flow sintering, which is performed at much lower temperatures than those required for remelting, is evidently favored and constitutes the fundamental processing step for waste-derived materials, such as glass matrix composites (to be used as alternatives to natural stones) [14] and, more importantly, glass foams (to be used for thermal and acoustic insulation) [15,16,17].

Sintering may also be a fundamental step for products with a not-stochastic porosity [18,19], such as scaffolds fabricated by additive manufacturing technologies starting from glass slurries. Dasan et al. [20] have recently discussed the manufacturing of three-dimensional translucent scaffolds, designed as supports for photocatalysts or parts of optical sensors (to detect noxious gases), by stereolithography as a reuse strategy for clear glass used in LCD displays.

With the development of ‘niche’ products, which have unprecedented characteristics but a well-recognizable utility, open-loop recycling is intended to become ‘upcycling’, i.e., provide extra revenues. This may be favored by specific features of the reused glass. In the case of LCD glass, the translucency of the sintered scaffold is based on a nearly full densification, with no concurrent devitrification, maximizing the optical quality of fired pieces. This in turn depends on the softening of glass at relatively high temperature, enabling the complete thermal degradation of organic binders before densification while avoiding the trapping of any pyrolytic residua [20]. A quite distinctive feature of pharmaceutical glass, explored in the present paper, is related to its sensitivity to alkali activation. Being practically calcium-free and rich in both B_2_O_3_ and Al_2_O_3_, alkali attack and the condensation of reaction products does not yield calcium silicate hydrated (C-S-H) gels, as in the case of common soda-lime glasses, but more stable semi-crystalline zeolite-like gels, resembling those formed in more established alkali-activated materials such as ‘geopolymers’ [17].

The formation of zeolite-like gels is promising in the perspective of the adsorption of organic molecules, such as industrial dyes and related compounds, recognized as key contaminants of waters. The adsorption of dyes is controlled by the overlapping contributions of the microstructure (depending on the surface area and porosity) and surface functional groups [18,21]. From this perspective, alkali-activated materials show a great potential [22,23].

The present paper investigates a novel combination of low-cost masked stereolithography with alkali activation for the manufacturing of recyclable porous monoliths [24], to be inserted in filtering devices. Compared to powder beds, monoliths, as unitary structures, can make the replacement of the filters easier upon saturation [24]. Foams represent a general solution [25], but attention is also paid to components with a non-stochastic porosity, such as honeycombs [26].

Compared to foams, honeycombs may lead to a lower pressure drop when dynamically filtering large volumes of water [27], but straight channels impede an intensive fluid-solid sorbent interaction. Components with a regular but tortuous structure, like the one offered by gyroids. (Cellular bodies defined by the packing of helicoidal channels, separated by curved membranes [20], effectively maximize the contact interface [28].) We will show that the contact interface can be enhanced by alkali activation of glass. Commercial adsorption processes generally use granular or pelleted materials, and the idea of using an adsorbent material in a monolith form is relatively recent.

## 2. Materials and Methods

### 2.1. Materials and Reagents

Colorless pharmaceutical glass waste, with the composition (71.7 SiO_2_, 9.8 B_2_O_3_, 6.7 Al_2_O_3_, 6.5 Na_2_O, 1.3 K_2_O, 1.1 CaO, 0.7 BaO, 0.02 TiO_2_, 0.02 Fe_2_O_3_, 0.01 SO_3_ in wt.%) [14], was used. Commercially available plant-based photocurable resin, made mainly from soybean plant, which is BPA-free and purchased from ELEGOO, China’s Silicon Valley, Shenzhen, was used as a UV photosensitive (405 nm) resin. Polyethylene Glycol (PEG) 400 (Sigma-Aldrich, Schnelldorf, Germany) was applied as a dispersing agent.

### 2.2. Synthesis and Procedures

Colorless pharmaceutical glass waste was crushed via ball milling and sieved below 40 µm. Fine glass powders were suspended in a plant photocurable resin, with 5 wt.% of Polyethylene Glycol (PEG). Plant resin and PEG were first homogenized at 400 rpm for 4 min, after which pharmaceutical glass was added with a solid loading content of 55 wt.%. The mixture was then homogenized at 2000 rpm for 10 min. Gyroid structures were printed by masked stereolithography (Prusa SL1S, Prusa Research a.s., Prague, Czech Republic).

The printer operated in the visible light range between 400 and 500 nm, with a layer thickness of 50 µm (exposure time 4 s for each layer), using the model of gyroids with 85% porosity in the form of cubic blocks with dimensions of about 10 mm × 10 mm × 10 mm. The adopted geometrical models (STL, Standard Triangulation Language) were used from a preliminary computational study by the Rhinoceros 6 program package (Robert McNeel & Associates, Seattle, WA, USA) [29].

After debinding at 330 °C for 12 h (heating rate 0.5 °C/min) followed by 600 °C for 5 h (the same heating rate), the printed green objects were fired at 680 °C and 730 °C for 1 h. The sintered objects were immersed in 2.5 M NaOH for 1 h to initiate the alkali activation, then dried at 75 °C for 24 h.

The fabricated glass objects were immersed for 5 h into 50 mL of methylene blue with an initial concentration of 50 mg/L, to investigate the adsorption efficiency of printed structures. The adsorption efficiency is calculated by applying Equation (1), where *C*_0_ represents the initial concentration of methylene blue (mg/L), and *C_e_* is the equilibrium concentration:

(1) $$\mathrm{The}\;\mathrm{adsorption}\;\mathrm{efficiency}=\frac{\left(C_{0\;}-C_{e}\;\right)}{C_{0\;}}*\;100\;$$

The structures fired at two different temperatures had the same weight of 0.33 g. The recyclability and the stability of the samples were investigated by heating the samples to 250 °C. 

### 2.3. Material Characterization

The obtained samples were examined by XRD before and after activation using an X-ray diffractometer Bruker AXS at room temperature using Cu-Kα radiation (λ = 1.5405 Å) and a scanning speed of 0.05°/min in the 2θ (Bragg angle) range from 10° to 70° and 40 kV/40 mA to detect the crystallized phases. In addition, the absorption infrared spectra of glass particles were measured using FT/IR-4200 Fourier Transform Infrared Spectrometers by JASCO (Easton, MD, USA). 

The microstructure of the printed pharmaceutical glass samples was examined after sintering at two different temperatures (680 and 730 °C), before and after activation, using scanning electron microscopy (FEI Quanta 200 ESEM, Eindhoven, The Netherlands) equipped with EDS. 

The densities were determined using a helium gas pycnometer (Anton Paar, Ultrapyc 3000, Graz, Austria). A stainless-steel ball (1.0725 g) was used as a calibration standard, with 7 readings for the calibration. The total porosity (*P*) was calculated by applying Equation (2), where $\rho_{geometrical\;}$ is the geometrical density and $\rho_{true}$ is the true density of the material:

(2) $$P\;\left(\%\right)=1-\frac{\rho_{geometrical}}{\rho_{true}}*100$$

The specific surface area was measured by N_2_ physisorption at −196 °C (ASAP 2010, Micromeritics, Norcross, GA, USA). The printed glasses were degassed at 150 °C, and the specific surface area was calculated in the relative pressure (p/p_0_) range between 0.05 and 0.30 by applying the Brunauer–Emmett–Teller (BET) multipoint method.

The compressive strength of the fired objects was measured using a universal testing machine (Quasar 25, Galdabini S.p.a., Cardano al Campo, Italy) operating at a cross-head speed of 0.5 mm/min. 

The remaining concentration of the dye after the adsorption was determined by measuring the absorbance of the solution at 664 nm (λmax) using a UV–VIS spectrophotometer (Jasco V-650, USA). 

## 3. Results and Discussion

The masked stereolithography technique used in this work offers an interesting trade-off between high precision manufacturing and cost minimization. In this technology, thin layers of photosensitive resin, deposited on an FEP (fluorinated ethylene propylene) plastic film, are selectively cured and bound to the printing head (moving upwards) by light passing through an underneath LCD screen. Light from a LED array only passes through the white pixels on the display, curing a projection area, layer by layer [30]. Using an LCD display as a ‘light filter’ makes masked stereolithography more cost-effective than digital light processing (DLP) based on the use of a projector [31].

**Figure 1:**
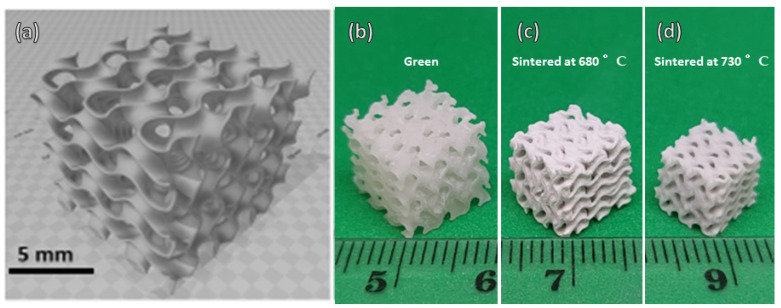
(**a**) Reference three-dimensional gyroid model; (**b**) printed gyroid before debinding and sintering; (**c**) gyroid after firing at 680 °C; (**d**) gyroid after firing at 730 °C.

The use of photocurable acrylates loaded with glass powders has an impact on the printing resolution compared to unfilled acrylates. Solid particles scatter light [32], not only altering the curing depth but also the curing time. Light does not simply cure the resin in the theoretical projection area (corresponding to a horizontal cross-section of the object), but it also propagates to the surrounding volume. This means that in a cellular body, the solid struts are enlarged and the overall porosity is reduced when compared to the model. A gyroid model with abundant porosity (85 vol%), shown in Figure 1, was intentionally applied to balance the scattering-induced coarsening. Table 1 shows that the porosity after printing (Figure 1b) was much reduced, but it still remained substantial (66 vol%).

Light scattering is not the only factor affecting the geometry of scaffolds during the printing of glass suspensions. In fact, the sintering mechanism of glass is particularly delicate: the extensive viscous flow of softened glass may completely degrade the cellular structure created by printing, with highly porous scaffolds transformed into smooth glass beads [33]. An acceptable balance between densification (joining of adjacent particles) and limited coarsening is typically offered by glass crystallization (the increase of viscosity produced by the precipitation of crystals ‘freezes’ the flow), but only for selected compositions [33]. In the present case, where the glass used was particularly stable against crystallization [14], the coarsening had to be controlled by the careful selection of the firing temperature.

**Figure 2:**
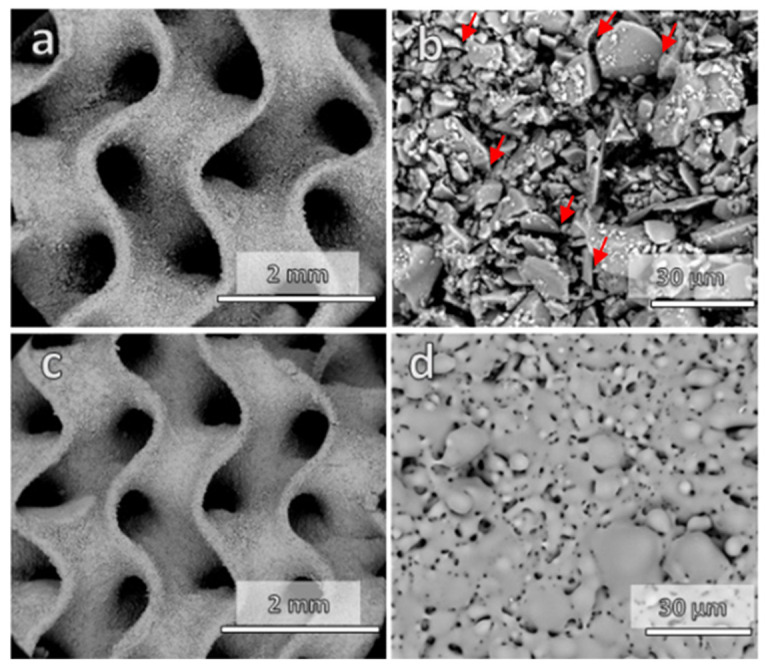
Microstructural details of gyroid scaffolds: (**a**,**b**) after firing at 680 °C; (**c**,**d**) after firing at 730 °C.

In general, the dilatometric softening point (Td) is recognized as the minimum temperature for viscous flow sintering [14], only leading to substantial densification if a pressure is applied simultaneously. Starting from a glass with Td = 650 °C (as determined by Bernardo and Scarinci [14]), a first series of sintering experiments at 680 °C (Td + 30 °C) was expected to only result in partial sintering. This is confirmed by the data in Table 1, especially the overall almost completely open porosity (~75%). Debinding and firing resulted in relatively thick (Figure 2a) but permeable struts, as documented by the microstructure in Figure 2b, which shows angular glass particles connected only by necks (marked by arrows in Figure 2b).

The main drawback of the low sintering temperature, implying incomplete sintering, was the low mechanical strength (<1 MPa, Table 1). To enhance the mechanical strength, the firing temperature was increased to 730 °C. This did not cause any significant degradation of the gyroid structure (Figure 2c), except for significant shrinkage (from 15% after firing at 680 °C, to 28%). A marked progress in densification was achieved, but at the same time the solid walls of the gyroid structures maintained an abundant and mostly open porosity (Figure 2d). The crushing strength increased significantly, up to 4 MPa. 

**Figure 3:**
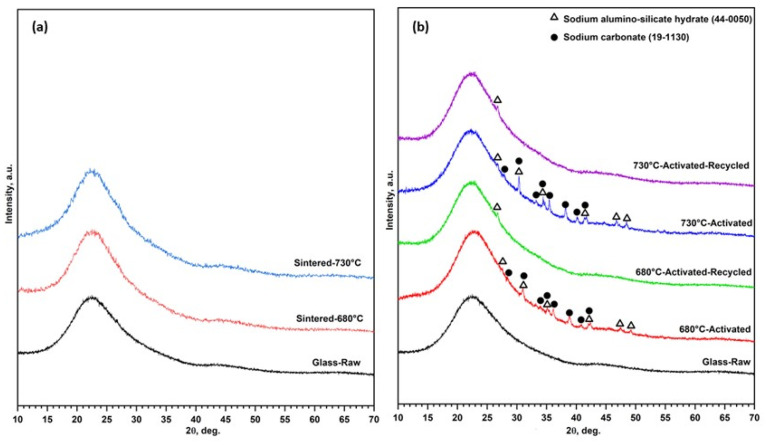
(**a**) XRD of glass sintered at 680 and 730 °C; (**b**) XRD of activated and recycled activated glass sintered at 680 and 730 °C.

**Figure 4:**
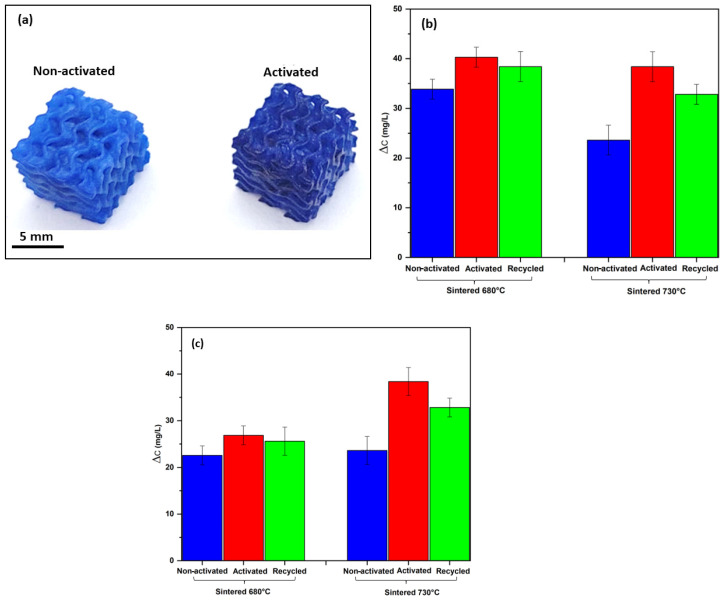
(**a**) Adsorption of methylene blue of activated and non-activated printed gyroid fired at 730 °C; (**b**) the uptake of methylene blue by the same weight of non-activated, activated and recycled gyroids; (**c**) the uptake of methylene blue by the same volume of non-activated, activated and recycled gyroids.

The preservation of the cellular structure was attributed to the relatively low sintering temperatures. The scaffolds fired at 680 °C and 730 °C without activation remained X-ray-amorphous (Figure 3a). The fired gyroids could be used as sorbents (Figure 4a, ‘non-activated’). Due to the lower porosity and consequently lower surface area, samples fired at 730 °C were less efficient than those fired at 680 °C (Figure 4b, ‘non-activated’). However, sintering was not the only parameter to consider. The adsorption of methylene blue from the solution could be enhanced by the alkali activation (Figure 4a).

**Figure 5:**
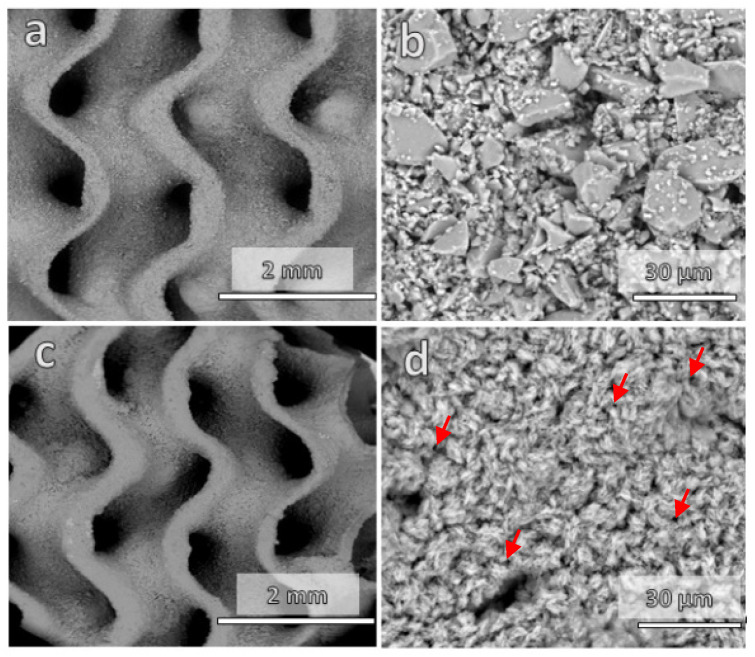
Microstructural details of activated gyroid scaffolds: (**a**,**b**) fired at 680 °C; (**c**,**d**) fired at 730 °C.

As observed by Rincon Romero et al. [17], the glass used in this study is sensitive to alkaline attack. After immersion in NaOH solution, extraction and drying, the surfaces of fired objects were coated with a uniform, partially crystallized xerogel layer (Figure 5). The formation of xerogel was attributed to the partial dissolution of the glass matrix, precipitation of corrosion products and interaction with the atmosphere. The X-ray powder diffraction confirmed the formation of a zeolite phase (sodium alumino-silicate hydrate, sodalite, 4Na_2_O·3Al_2_O_3_·6SiO_2_·3H_2_O, PDF 44-0050) coupled with a sodium carbonate phase (Na_2_CO_3_, PDF 19-1130), as shown in Figure 3a. 

After activation, the specific surface area increased by 10 and 40% for glasses sintered at 730 °C and 680 °C, respectively, as shown in Table 1, and the adsorption capability increased significantly (Figure 4b). The surface functions of silica glass, an oxide adsorbent, are related to the presence of silanol (Si-OH) groups. At a sufficient concentration, the presence of these groups makes the surface hydrophilic. The OH groups act as the centers of molecular adsorption during their specific interaction with the adsorbates, which form a hydrogen bond with the OH groups; or, more generally, they undergo a donor–acceptor interaction [34]. Figure 4c shows the adsorption capacity of methylene blue through the same volume of non-activated, activated and recycled gyroids. The gyroids sintered at 730 °C exhibited a higher capacity for adsorption of the dye, accompanied by better mechanical properties (Table 1). 

The partially charged activated glass interacts electrostatically with the cationic methylene blue species, resulting in a more pronounced adsorption of the dye [35]. Moreover, the formation of channels in the sodium aluminum silicate hydrate phase (marked by arrows in Figure 5d) increases the adsorption capacity. The carbonate phases also act as an active adsorbent for dye adsorption [2].

The use of gyroid structures was attractive for the dye adsorption due to their versatile architecture (adaptable pore size and porosity) along with their strong mechanical properties and mass transport (permeability and diffusivity) properties [36]. The successive adsorption of methylene blue dye on the glass surface may be considered to be the result of a combination of the chemistry of the surface and the applied 3D porous structure. Chakrabarti and Dutta demonstrated that the process of adsorption of methylene blue dye by the glass adsorbent consisted of its sorption at the surface followed by diffusion [37].

The non-activated glass showed a lower adsorption capability due to the less active surface, which lacks porous zeolitic phases (higher surface area). 

The reported adsorption efficiency for methylene blue by glasses and 3D-printed materials is presented in Table 2. It should be noted that Kinoshita et al. showed that glass fibers did not adsorb methylene blue at all [38]. Applying a congo-red dye, they found a considerable adsorption due to the capacity for ion exchange with calcium ions. 3D-printed glass is superior to porous ceramic filters in terms of adsorption efficiency and regeneration performance. In particular, the adsorption efficiency of 3D-printed glass was close to the other printed materials, which were in the range of 83–93%. 

The printed glass adsorbent can be easily separated from the dye solution without centrifugation, filtration or magnetic separation. The possibility of using glasses as adsorbents makes the process attractive, as the waste generated from used glasses can also have a potential use.

**Figure 6:**
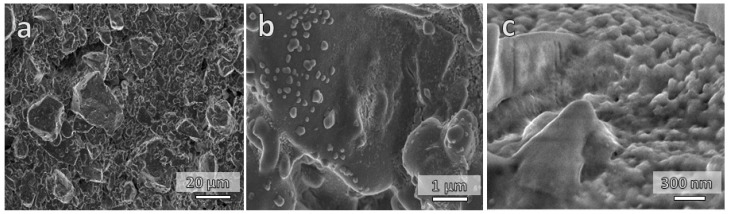
Microstructural details of gyroid scaffolds: (**a**) fired at 680 °C; (**b**) the recycled gyroid fired at 680 °C; and (**c**) the recycled gyroid fired at 730 °C.

To verify the recyclability of the activated sintered gyroids, the scaffolds were subjected to three consecutive adsorption cycles to evaluate the preservation of their adsorption capacity. After each adsorption cycle, the scaffolds were heated at 250 °C for 24 h for reuse. The zeolite phase did not degrade as the result of the heat treatment (Figure 3b). Despite the removal of the sodium carbonate phase, the uptake of methylene blue by recycled sintered samples remained substantial (Figure 4b). Figure 6a confirms the lack of firing temperature for complete sintering. The regeneration performance of the printed objects was quite close to the original objects in terms of the adsorption efficiency due to several reasons: the complete degradation of MB during the thermal regeneration, the slight change in the mass of the adsorbents, and the non-degradable sodalite phase, which worked as an adsorbing center. Figure 6b,c show the remains of the sodalite structure on the recycled gyroids fired at 680 °C and 730 °C. At the higher magnification, the microstructure shows a rough texture that could enhance the adsorption capacity compared to the homogenous, smooth surface of the as-sintered glass.

## 4. Conclusions

Glass from discarded pharmaceutical vials may be conveniently reused for the fabrication of a new generation of monolithic sorbents for dye removal. Masked stereolithography, combined with a careful selection of sintering conditions, enables the fabrication of components with a tortuous porosity, maximizing the interaction of methylene blue solution with the solid matrix. The interaction is enhanced by alkali activation, exploiting the sensitivity of the used glass and leading to the development of a surface gel comprising both soluble sodium carbonate and insoluble sodalite. The latter phase was responsible for the preservation of the adsorption capacity of tested scaffolds after multiple adsorption/heat treatment cycles. 

## Figures and Tables

**Table 1:** Physical and mechanical properties of activated and non-activated gyroids.

Samples	Shrinkage (%)	Geometrical Density (g/cm^3^)	Apparent Density (g/cm^3^)	True Density (g/cm^3^)	Open Porosity (%)	Closed Porosity (%)	Total Porosity (%)	BET (m^2^/g)	Compressive Strength (MPa)
Green	-	0.54 ± 0.02	1.6 ± 0.01	-	66 ± 1	-	-	-	-
Glass 680 °C	15 ± 2	0.54 ± 0.02	2.28 ± 0.03	2.38 ± 0.02	76 ± 2	2 ± 1	78 ± 1	0.7	0.8 ± 0.2
Glass 680 °C Activated		0.56 ± 0.01	2.31 ± 0.03	2.41 ± 0.02	76 ± 2	1 ± 1	77 ± 1	1.1	0.7 ± 0.1
Glass 730 °C	28 ± 3	0.84 ± 0.03	2.24 ± 0.02	2.33 ± 0.03	62 ± 1	2 ± 1	64 ± 1	0.6	4.0 ± 0.2
Glass 730 °C-Activated		0.92 ± 0.02	2.26 ± 0.03	2.46 ± 0.05	60 ± 1	3 ± 1	63 ± 1	0.7	4.2 ± 0.1

**Table 2:** A comparison of adsorbents with their adsorption efficiency for methylene blue dye.

Adsorbent	Adsorption Efficiency (%)	Ref.
Borosilicate glass	70	[39]
Porous ceramic filter	72.1	[40]
This work	81	
3D-printed geopolymer	83.6	[21]
3D-printed chitosan/nano-TiO_2_	84.9	[41]
3D-printed magnetic cellulose	88.5	[42]
3D-printed carbon	93	[43]

## Data Availability

The data presented in this study are available on request from the corresponding author.
